# Combined application of single-energy metal artifact reduction and reconstruction techniques in patients with Cochlear implants

**DOI:** 10.1186/s40463-020-00462-1

**Published:** 2020-09-09

**Authors:** Fanqin Wei, Jiahui Li, Chunxiang Zhou, Yun Li, Xianren Wang, Bixue Huang, Qiyang Sun, Guanxia Xiong

**Affiliations:** 1 Department of Otorhinolaryngology Head and Neck Surgery, the First Affiliated Hospital, Sun Yat-sen University, 2nd Zhongshan Road 58#, Guangzhou, 510080 Guangdong PR China; 2 Institute of Otorhinolaryngology Head and Neck Surgery, Sun Yat-sen University, Guangzhou, 510080 Guangdong PR China; 3 Guangzhou key Laboratory of Otorhinolaryngology, Guangzhou, 510080 Guangdong PR China; 4 Guangzhou Women and Children’s Medical Centre, Guangzhou, 510623 Guangdong PR China

**Keywords:** SEMAR; electrode, Image quality, Computed tomography

## Abstract

**Background:**

The purpose of this study was to develop an effective method of reducing metal artifacts in cochlear implant (CI) electrodes.

**Methods:**

The temporal bones of 30 patients (34 ears) after CI were examined with 320-detector row computed tomography, which was evaluated by two senior radiologists using a double-blind method. Noise, artifact index, signal-to-noise ratio, and the subjective image quality score were compared before versus after using single-energy metal artifact reduction (SEMAR). The electrode position, single electrode visibility, and electrode count were evaluated using SEMAR combined with either multi-planar reconstruction (MPR) or maximum intensity projection.

**Results:**

The two radiologists’ measurements had good consistency. SEMAR significantly reduced the image noise and artifacts index and significantly improved the signal-to-noise ratio and subjective image quality score (*P* < 0.01). The combination of SEMAR with MPR was conducive to accurate assessment of electrode position and single-electrode visibility. The combination of SEMAR with MIP facilitated accurate and intuitive matching of the assessed electrode count with the number of electrodes implanted during the operation (*P* = 0.062).

**Conclusion:**

SEMAR significantly reduces metal artifacts generated by CI electrodes and improves the quality of computed tomography images. The combination of SEMAR with MPR and maximum intensity projection is beneficial for evaluating the position and number of CI electrodes.

## Background

Cochlear implants (CIs) are currently the most effective treatment for patients with severe-to-profound sensorineural hearing loss, and they have been widely used worldwide. Hearing outcomes after CI have been the most important issue to patients and doctors because they can be affected by many factors. In addition to the cause, duration, and extent of hearing loss and the development of speech function before implantation, the electrode position in the cochlea can also play an essential role in the postoperative effects of CIs [1–3]. Because electrodes cannot be observed in the cochlea by naked eye after implantation, methods to evaluate the electrode position after CI are needed.

The main methods to perform such evaluation are currently X-ray, computed tomography (CT), and other imaging examinations. Among them, X-ray cannot show the structural relationship between the electrodes and the cochlea or their movement in the cochlea [4]. CT has good spatial resolution, contributing to its accuracy in evaluation of electrode implantation. However, CT has higher levels of metal artifacts, which significantly affect the image quality [5–7]. Therefore, methods to reduce or remove artifacts around metal implants are clinically important. Recently, the application of energy spectrum CT [8] and various post-processing technologies [9] after artifact removal has provided some solutions, but the results of such artifact removal remain unsatisfactory.

Single-energy metal artifact reduction (SEMAR) is a new method of metal artifact removal from CT that is time-saving, easy to operate, and reduces the radiation dose [10]. Evaluation of SEMAR has focused on artifacts generated by larger metal implants, such as hip joint, spine, and dental metal implants [11, 12]. However, the evaluation of SEMAR in cochlear implants has not been reported. We seek to determine how effective SEMAR is at improving metal artifacts produced by cochlear electrodes and how to apply SEMAR to electrode evaluation after CI.

In this study, we retrospectively analyzed CT examination results from patients after CI to evaluate the effects of SEMAR on electrode metal artifact removal. The position and number of electrodes were also displayed after SEMAR was combined with multi-planar reconstruction (MPR) and maximum intensity projection (MIP) to assess the methods’ potential application in electrode evaluation after CI.

## Materials and methods

### Patient population

We included patients who underwent routine 320-row CT (Aquilion ONE, Toshiba Medical Systems, Otawara, Japan) postoperative examination after CI at our institution between August 2018 and February 2019. All patients were included only once (i.e., repeated CT examinations were excluded). The patients underwent CT examination within 1–4 days after CI. During the examination, the patients were placed in supine position with bilateral symmetry. If the patient failed to cooperate with the examination, 10% chloral hydrate was taken orally, and the patient was scanned again after falling asleep.

### Objective assessment of image quality

To objectively evaluate image quality, the images acquired on a Toshiba 320-row multi-slice spiral CT were exported to a Vitreacore 6.9.87.1 (Minnetonka, MN, USA) post-processing workstation. A region of interest (ROI) was placed in the bottom of the cochlea, and we measured the number of Hounsfield Units (HU) and its standard deviation (SD) in that ROI. The measurement location and scope were consistent within the same patient and had an area of approximately 5–6 mm^2^. The SD of the signal within the ROI was set as the image noise. The HU value of the cochlear canal bone was measured when the electrode was rotated at the bottom of the cochlea. This measurement was taken three times, and the mean value was adopted. The CT and SD values of the same tissue with no artifact layer in the preoperative CT image were selected as reference**.** The artifacts index (AI) was calculated by the following formula: AI= $\sqrt{{\mathrm{SD}}_{\mathrm{artifact}}^2-{\mathrm{SD}}_{\mathrm{reference}}^2}$ [12]. The signal-to-noise ratio (SNR) was then calculated by dividing the mean attenuation number by the corresponding SD (SNR = CT / SD) [13]. The quantitative values before and after artifact removal were compared. We also applied MIP to count the electrodes directly.

### Subjective assessment of image quality

To evaluate the subjective image quality, two senior radiologists (with 20 and 27 years of experience) who were blinded to the patients’ identities and the image grouping independently evaluated the images both before and after artifact removal. We set the images without metal artifact removal as Group A and the images after metal artifact removal as Group B. To evaluate if there was any intra-observer variability, the two radiologists scored the images twice at different times. The image quality evaluation before and after artifact removal mainly focused on the display of the electrodes and cochlea. The rating of the MPR images focused on the electrodes’ specific placement, whereas evaluation of MIP images was geared towards observing the overall situation of the electrode. All of the evaluation standards were referenced three-point (0–2) scales, as described by Bartling et al. and Tobias Struffert et al. [14, 15]

The cochlea was rated on a three-point scale, where 0 = severely compromised by artifacts or blurred; 1 = slightly compromised by artifacts or blurred; and 2 = not compromised at all.

Artifacts (blurring, beam hardening) were rated as: 0 = images severely compromised; 1 = minimal artifacts, minimal blurring, but evaluation of inner ear structures and electrode is possible; 2 = without relevant artifacts.

The bony structures’ homogeneity was rated as: 0 = obvious noise; 1 = minimal noise; 2 = homogeneous bony structures, noise barely seen.

The position of the implant within the cochlea concerning the scala vestibuli and scala tympani was rated as: 0 = the electrode is within the cochlea, no other details can be seen; 1 = the position of the electrode relative to the modiolus can be suspected in some parts; 2 = the position of the electrode can be determined relative to the modiolus.

The visibility of single electrodes was evaluated as: 0 = not distinguishable; 1 = blurred, suspected single electrodes can be identified; 2 = single electrodes clearly visible.

### Scanning method and related image-processing methods

**Fig. 1:**
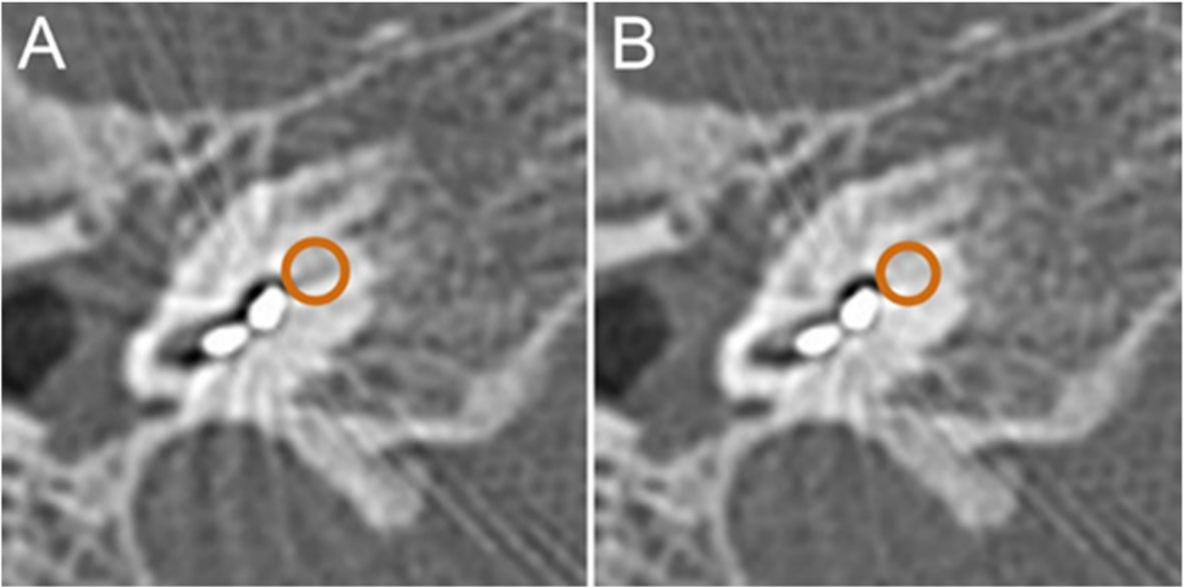
**a** ROI on a CT image without metal artifact removal. **b** ROI on a CT image with metal artifacts removed. ROI: regions of interest

The localization image was obtained using dual scan mode scanning, and the scanning range was adjusted according to the localization image to range from the tip of the mastoid to the upper edge of the implanted signal receiver. In S&V mode, one layer was swept within the scanning range, and two ROIs were set inside. The high-resolution reconstruction method was adopted, and the scanning parameters were as follows: 120 kVs, 250 mAs, 1-s rotation time, 0.5-mm reconstruction thickness, 0.1-mm reconstruction interval, 512 × 512 matrix, and 50-mm field of view. After the image was transferred to the workstation, two groups of diagrams were obtained for Group A (without metal artifacts removed) and Group B (after SEMAR-based metal artifact removal) (Fig. 1). Then, two reconstruction techniques were applied to Group B: MPR and MIP. The reconstruction method was as follows: transmit the overlapping magnified reconstruction image to a Vitreacore 6.9.87.1 workstation, import it in “Inspace” mode, select the threshold range of − 1000 to + 1000 HU to display the inner ear structure, and then add a threshold range of 2500–5000 HU to display the electrode.

### Statistics

Statistical analysis was performed with SPSS (version 25.0). Two independent-samples *t* tests were used for inter-group comparison. Enumeration data were compared using χ^2^ tests. Differences were deemed statistically significant at *P* < 0.05.

## Results

### Patient

A total of 30 follow-up CT examinations (34 ears) were included in the SEMAR reconstructions. The median patient age was 4.5 years (range, 1–64 years), and the patients included 12 female (13 ears) and 18 male (21 ears) patients. All patients presented with binaural severe or profound sensorineural hearing loss and met the criteria for CI evaluation. The patient and electrode characteristics are summarized in Table 1. Formal informed consent was not deemed necessary for this retrospective study by the medical ethical board.

**Table 1:** Patient and electrode characteristics

Patient characteristics (*n* = 34 ears)	
Median age	4.5
Male	21 (62%)
Female	13 (38%)
Normal cochlea development	29 (85%)
Hypoplasia of the cochlea (CH3)	2 (6%)
Cochlea ossification class III	1 (3%)
Cochlea ossification class II (Sequela of meningitis)	2 (6%)
Secondary cochlear implantation	1 (3%)
Combi 40+ MED-EL^a^ cochlea implantation (12 electrodes)	24 (71%)
Advanced Bionics^a^ cochlea implantation (16 electrodes)	10 (29%)
Cochlear implantation in right ear	22 (65%)
Cochlear implantation in both ears	4(13%)

^a^Both types of cochlear implants are made of platinum and iridium

### Subjective assessment of SEMAR images

**Fig. 2:**
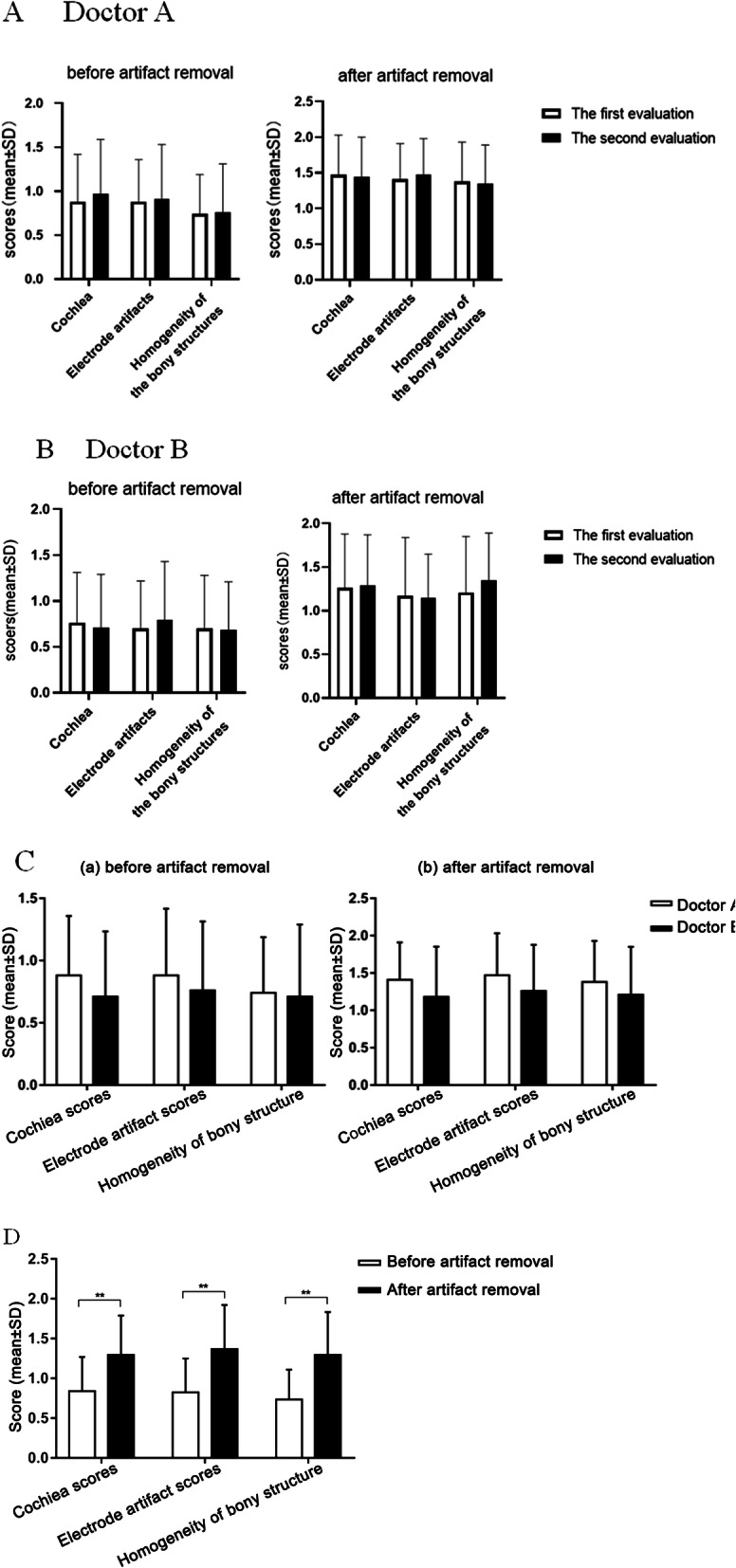
Consistency analysis and scoring results of the subjective scoring before and after artifact processing. **a** shows the results of doctor A’s two evaluations before and after the removal of the artifact, *P*> 0.05, suggesting that the differences between the two evaluations are small. **b** shows that the results of Doctor B are the same as those of Doctor A. **c** shows no significant difference was found between the three subjective scores between before and after artifact removal (*P* > 0.05), indicating good inter-observer consistency. Because of this consistency, we grouped the two doctors’ scores into a single dataset

**Fig. 3:**
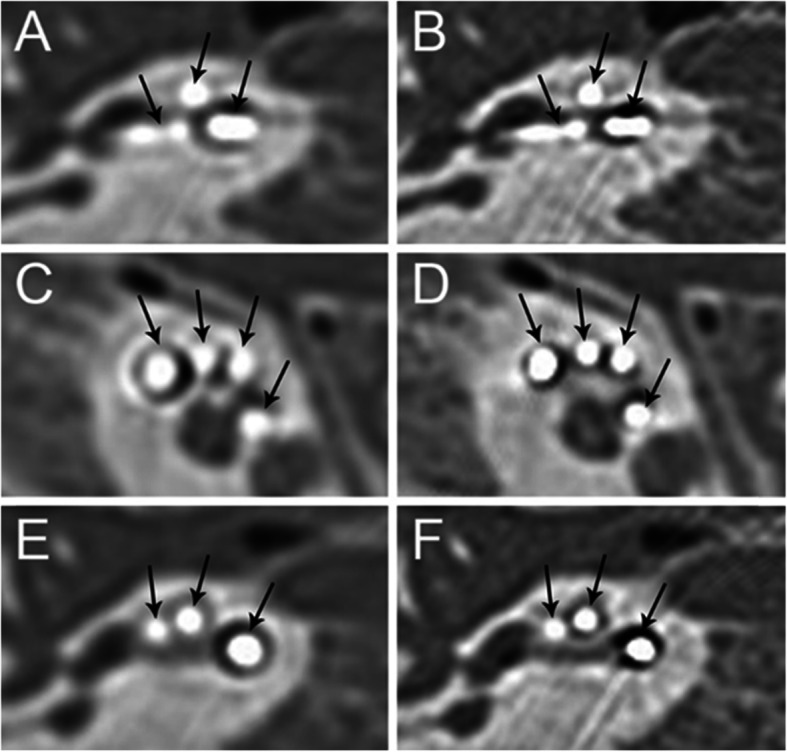
CT images of cochlear implants before and after using SEMAR. The image is greatly affected by electrode artifacts before using SEMAR, as part of the cochlea is obscured by the artifacts, and the electrode condition is displayed unsatisfactorily (**a**, **c**, **e**). The electrode artifacts in the image are reduced after using SEMAR, which allows clear observation of the corresponding structure and specific conditions of electrodes (**b**, **d**, **f**). The arrows in the figure indicate electrodes

To explore the application of SEMAR to electrode evaluation after CI, two radiologists subjectively graded the cochlea, electrode artifacts, and bone structure uniformity on CT images. We found that before and after the application of SEMAR, intra-observer reliability between different times was high (Fig. 2, Tables 2 and 3), and no significant difference was found between the two radiologists’ scores for the cochlea, electrode artifacts, or bone structure uniformity, indicating good inter-observer consistency (Fig. 2). The Group A images were greatly affected by electrode artifacts: part of the cochlea was obscured by the artifacts, and the visibility of the electrode conditions was poor. However, electrode artifacts were reduced in Group B images, which allowed clear observation of the conditions of the cochlea and electrodes (Fig. 3). The scores for cochlea, electrode artifacts, and bone structure uniformity were significantly improved in Group B (*P* < 0.01), suggesting that SEMAR improves CT image quality after CI (Fig. 2).

**Table 2:** The analysis of intra-observer reliability with non-SEMAR. (*n* = 34 each) (x ± s)

	The first evaluation	The second evaluation	t	p
**Cochlea**				
Doctor A	0.88 ± 0.54	0.97 ± 0.63	− 0.90	0.37
Doctor B	0.76 ± 0.55	0.71 ± 0.58	0.57	0.57
**Electrode artifacts**				
Doctor A	0.88 ± 0.48	0.91 ± 0.62	−0.29	0.76
Doctor B	0.71 ± 0.52	0.79 ± 0.64	−1.14	0.26
**Homogeneity of bony structures**				
Doctor A	0.74 ± 0.45	0.76 ± 0.55	−0.37	0.71
Doctor B	0.71 ± 0.57	0.68 ± 0.53	0.33	0.74

**Table 3:** The analysis of intra-observer reliability after the application of SEMAR. (*n* = 34 each) (x ± s)

	The first evaluation	The second evaluation	t	p
**Cochlea**				
Doctor A	1.47 ± 0.56	1.44 ± 0.56	0.30	0.77
Doctor B	1.26 ± 0.62	1.29 ± 0.58	−0.30	0.77
**Electrode artifacts**				
Doctor A	1.41 ± 0.49	1.47 ± 0.51	−0.57	0.57
Doctor B	1.18 ± 0.67	1.15 ± 0.50	0.33	0.74
**Homogeneity of bony structures**	1.38 ± 0.55	1.35 ± 0.54	0.27	0.78
Doctor A	1.38 ± 0.55	1.35 ± 0.54	0.27	0.78
Doctor B	1.21 ± 0.64	1.35 ± 0.54	−1.15	0.26

### Quantitative assessment of SEMAR images

To evaluate the SEMAR images quantitatively and objectively, we measured the CT and SD values of the cochlear canal before and after artifact removal. Each of the objective evaluation scores (for noise, AI, and SNR) differed significantly between Groups A and B (*P* < 0.01): the image noise and AI of Group B were significantly lower than those of Group A, and the SNR was significantly higher in Group B. Thus, SEMAR reduced the magnitude of image artifacts and improved image quality significantly (Table 4).

**Table 4:** Objective analysis of the two groups’ image quality (*n* = 34 each) (x ± s)

	Non-SEMAR	SEMAR	t	p
CT value (HU)	1726.41 ± 54.96	1720.29 ± 28.65	0.65	0.519
Image noise (HU)	79.18 ± 35.40	58.94 ± 20.68	2.88	0.007
AI(HU)	61.72 ± 43.04	35.30 ± 31.21	3.06	0.004
SNR	23.39 ± 12.49	33.63 ± 14.96	−3.04	0.005

*HU* Hounsfield Units, *AI* Artifacts index, *SNR* Signal-to-noise ratio, *SEMAR* Single energy metal artifact reduction

### Evaluation of images processed by SEMAR combined with MPR or MIP

**Fig. 4:**
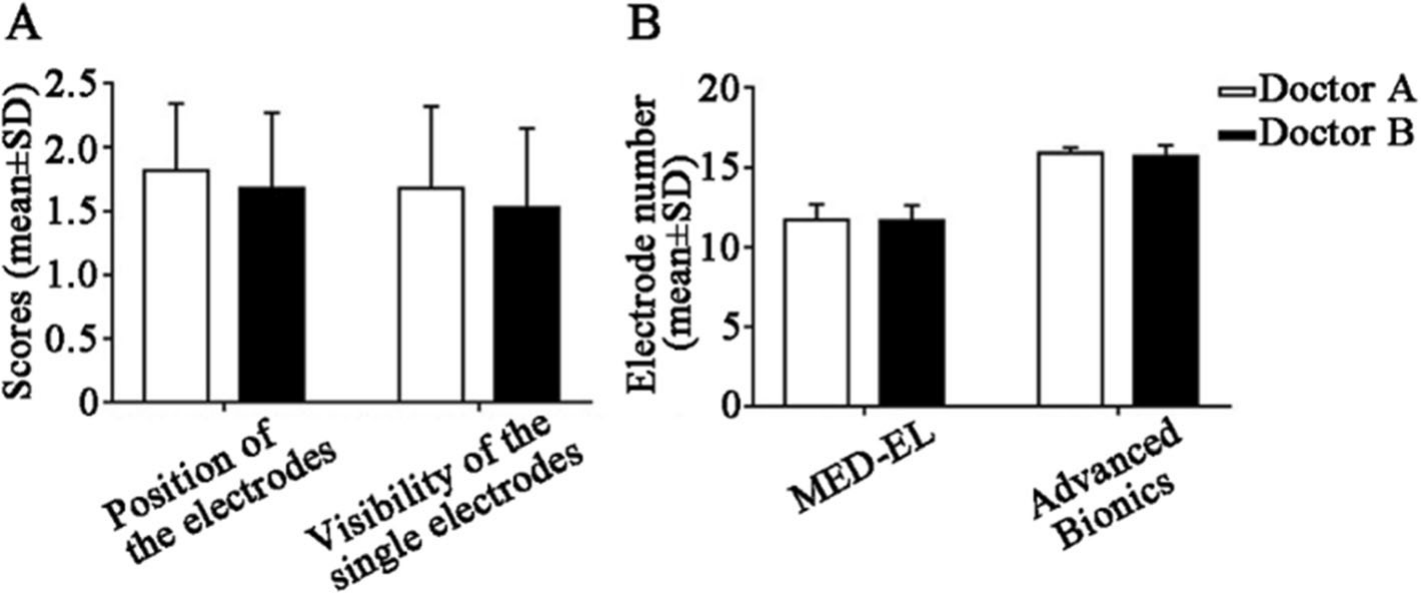
Consistency between two radiologists on scores of images processed by MPR and MIP. **a** The *P*-values of the relevant scores were all greater than 0.05, meaning that the two doctors’ scores were consistent. **b** Both radiologists counted the electrodes, and the agreement regarding both MED-EL and Advanced Bionics was unanimous

**Fig. 5:**
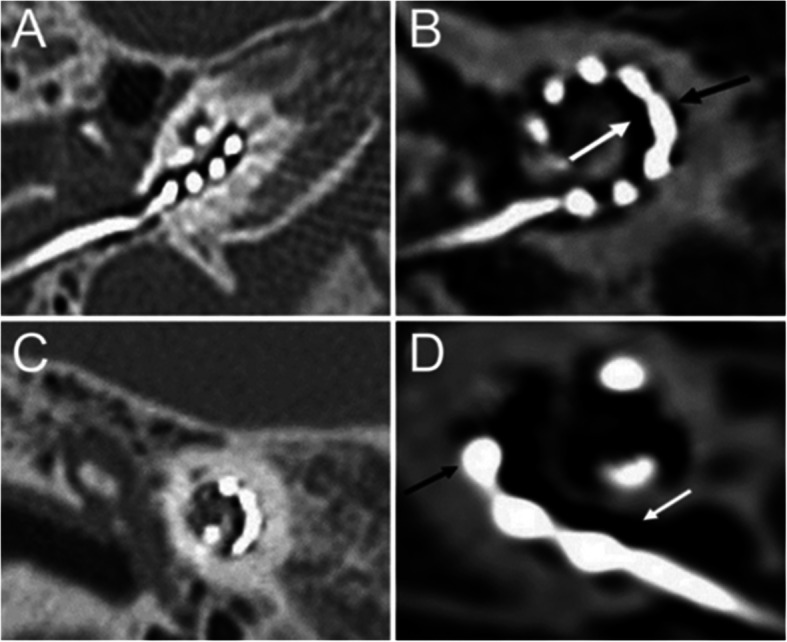
CT image of cochlea after using SEMAR combined with MPR. In the SEMAR CT image, the electrode is clearly located in the cochlea, it is difficult to evaluate whether the electrode is located in the scala tympani (**a**, **c**). Combining SEMAR with MPR enables clarification that the electrode is close to the cochlear wall. However, there is a clear spatial distance between the axes, thus the electrode is determined to be located in the scala tympani (**b**, **d**). The arrows indicate the electrode position

**Fig. 6:**
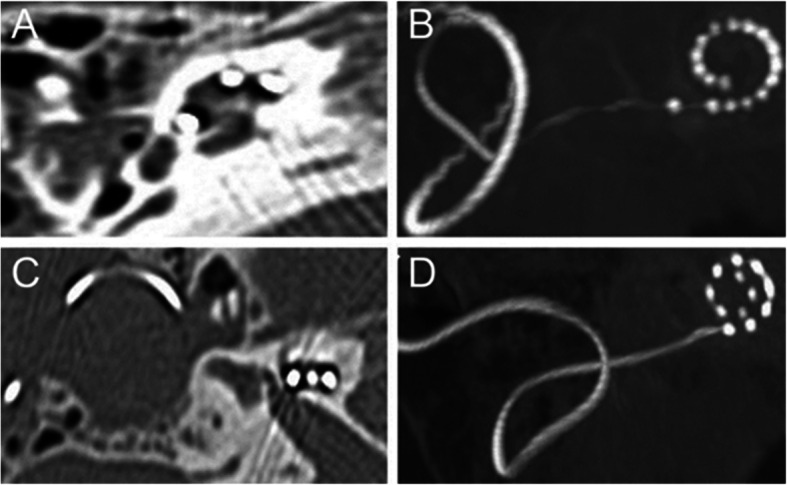
CT image of cochlea after using SEMAR combined with MIP. Counting electrodes using the SEMAR CT image requires superimposition of electrodes from different layers to count the electrodes in the cochlea, which requires complex operations (**a**, **c**). Using SEMAR combined with MIP enables us to directly count the cochlear electrodes in a single image. Advanced Bionics cochlear implantation: excluding the reference electrode (1) outside the cochlea, the number of electrodes inside the cochlea was 16 (**b**). Combi 40+ MED-EL cochlea implantation: 12 electrodes were counted (**d**)

To further evaluate the accuracy of electrode positioning in the cochlea, we performed MPR on the CT images after the SEMAR-based artifact removal. Furthermore, to count the number of electrodes in the cochlea, we combined MIP with SEMAR. The scores for electrode position, single-electrode visibility, and electrode count were consistent between the two radiologists (Fig. 4). We found that images processed with SEMAR combined with MPR clearly elucidated the inner ear’s fine structure, which is conducive to accurate assessment of the electrode position in the cochlea and its effect on the modiolus and bone spiral plate (Fig. 5). Furthermore, SEMAR combined with MIP clearly showed the overall electrode condition. Additionally, the electrodes could be counted accurately: the count matched the number of electrodes implanted during the operation (*P* = 0.062) (Fig. 6).

## Discussion

Postoperative imaging evaluation of CI has great value for clinical evaluation of the number, depth, and position of implanted electrodes [16]. Although the depth and number of implanted electrodes can be measured and counted during the operation, the possibility of rear electrode displacement cannot be excluded. The study of Rivaset and Fayad showed that it is optimal to observe the displacement of implanted electrodes in the first week after CI [17, 18]. This is because no fiber sheath or fixation cover grows within 1 week after the surgery. Therefore, complications such as shedding and twisting may occur, but generally, this does not degrade electrode performance or cause clinically visible discomfort. Postoperative imaging examination of CI is one of the main methods to determine electrode condition, especially among patients with poor hearing after CI. Therefore, comprehensive evaluation needs to be done to rule out problems with the cochlear electrodes after the operation.

Previous studies have indicated that high-resolution CT can facilitate identification of the inner ear’s fine structure and evaluation of the specific situation of electrodes in the cochlea after CI [19]. However, the artifacts caused by metal electrodes make the evaluation of postoperative complications and electrodes difficult. In this study, applying SEMAR significantly reduced image noise and AI and significantly improved the subjective scores of SNR and image quality, suggesting that SEMAR reduces metal artifacts of cochlear electrodes and improves image quality. However, SEMAR had no significant effect on the CT value of the bone cochlear canal bone (*P* > 0.05), which was different from the method’s results in other fields [11, 12]. Bamberg believed that the size of metal artifacts mainly depends on the thickness and material of the metal [20]. The soft tissues around the implant, such as muscle and fat, are the most disturbed by the artifacts. Previous research has mainly focused on the effects of SEMAR on the removal of artifacts of larger metal implants in the human body, and those studies measured the CT values of the soft tissues around the implants. Therefore, we consider that SEMAR may have a small effect on CT values of the inner ear because of the small volumes of the cochlear electrodes and the bone around them.

The inner ear’s structure is fine, with dimensions mostly on the millimeter scale, and therefore the choice of observation level greatly impacts the examination results. The conventional axial and sagittal orientations are not observed along the axis of the inner ear structure, which may introduce misinterpretation [21]. The structural identification of the inner ear used high-resolution CT, but conventional CT images can be adjusted to any level using MPR. Previous literature reported that double oblique MPR can fully display the temporal bone structure. Compared with axial and coronal images, the post-MPR images presented the inner ear structure more clearly and intuitively. At the same time, compared with the three-dimensional reconstruction method, the MPR method is more objective, less susceptible to human factors, and simpler to operate [22]. Some scholars believe that MPR can improve the detection rate and accuracy of inner ear deformities [23, 24]. In this study, we combined MPR with SEMAR and successfully showed that the electrodes in the investigated cases were all located in the cochlea (i.e., without incorrect implantation).

In post-processing of CT images, MIP is a method of projecting three-dimensional spatial data onto the visual plane [25]. We used MIP to project high-density electrodes onto the CT image plane, and this showed the overall electrode conditions clearly, so that the electrodes could be counted directly in a single image. The resulting count was consistent with the number of electrodes actually implanted during the operation (*P* = 0.062). Because the CIs used in our center are MED-EL and Advanced Bionics (12 and 16 electrodes respectively), there is a certain distance between the electrodes, and single electrodes are less affected by metal artifacts. Subsequent studies should include more types of implants to evaluate SEMAR combined with MPR/MIP using more electrodes and less electrode spacing.

## Conclusions

In summary, SEMAR can significantly reduce metal artifacts generated by CI electrodes and improve CT image quality. SEMAR combined with MPR and MIP can clearly, intuitively, and accurately display the shape, position, and number of electrodes in the cochlea, which is worthy of clinical application.

## Data Availability

All data are available upon request from the authors.

## Abbreviations

SEMAR: Single-Energy metal artifact reduction
CI: Cochlear implant
MPR: Multi-planar reconstruction
MIP: Maximum Intensity Projection
AI: Artifact index
SNR: Signal-to-noise ratio
ROI: Regions of interest

## References

[1] Aschendorff A, Kromeier J, Klenzner T, Laszig R (2007). Quality control after insertion of the nucleus contour and contour advance electrode in adults. Ear Hear.

[2] Finley CC, Holden TA, Holden LK, Whiting BR, Chole RA, Neely GJ, Hullar TE, Skinner MW (2008). Role of electrode placement as a contributor to variability in cochlear implant outcomes. Otol Neurotol..

[3] Teymouri J, Hullar TE, Holden TA, Chole RA (2011). Verification of computed tomographic estimates of cochlear implant array position: a micro-CT and histologic analysis. Otol Neurotol..

[4] Bettman RH, van Olphen AF, Zonneveld FW, Huizing EH (2003). Electrode insertion depth in cochlear implantees estimated during surgery, on plain film radiographs and with electrode function testing. Eur Arch Otorhinolaryngol.

[5] Wang G, Vannier MW, Skinner MW, Cavalcanti MG, Harding GW (1998). Spiral CT image deblurring for cochlear implantation. IEEE Trans Med Imaging.

[6] Whiting BR, Bae KT, Skinner MW (2001). Cochlear implants: three-dimensional localization by means of coregistration of CT and conventional radiographs. Radiology..

[7] van Wermeskerken GK, van Olphen AF, Graamans K (2009). Imaging of electrode position in relation to electrode functioning after cochlear implantation. Eur Arch Otorhinolaryngol.

[8] Bamberg F, Dierks A, Nikolaou K, Reiser MF, Becker CR, Johnson TR (2011). Metal artifact reduction by dual energy computed tomography using monoenergetic extrapolatio. Eur Radiol.

[9] Cahir JG, Toms AP, Marshall TJ, Wimhurst J, Nolan J (2007). CT and MRI of hip arthroplasty. Clin Radiol.

[10] Yasaka K, Kamiya K, Irie R, Maeda E, Sato J, Ohtomo K (2016). Metal artefact reduction for patients with metallic dental fillings in helical neck computed tomography: comparison of adaptive iter-ative dose reduction 3D (AIDR 3D), forward-projected model-based iterative reconstruction solution (FIRST) and AIDR 3D with single-energy metal artefact reduction (SEMAR). Dentomaxillofac Radiol.

[11] Sonoda A, Nitta N, Ushio N, Nagatani Y, Okumura N, Otani H, Murata K (2015). Evaluation of the quality of CT images acquired with the single energy metal artifact reduction (SEMAR) algorithm in patients with hip and dental prostheses and aneurysm embolization coils. Jpn J Radiol.

[12] Pan YN, Chen G, Li AJ, Chen ZQ, Gao X, Huang Y, Mattson B, Li S (2019). Reduction of metallic artifacts of the post-treatment intracranial aneurysms: effects of single energy metal artifact reduction algorithm. Clin Neuroradiol.

[13] Behrendt FF, Schmidt B, Plumhans C, Keil S, Woodruff SG, Ackermann D, Mühlenbruch G, Flohr T, Günther RW, Mahnken AH (2009). Image fusion in dual energy computed tomography effect on contrast enhancement, signal-to-noise ratio and image quality in computed tomography angiography. Investig Radiol.

[14] Bartling SH, Gupta R, Torkos A (2006). Flat-panel volume computed tomography for cochlear implant electrode array examination in isolated temporal bone specimens. Otol Neurotol.

[15] Struffert T, Hertel V, Kyriakou Y, Krause J, Engelhorn T, Schick B, Iro H, Hornung J, Doerfler A (2010). Imaging of cochlear implant electrode array with flat-detector CT and conventional multislice CT: comparison of image quality and radiation dose. Acta Otolaryngol.

[16] Vogl TJ, Tawfik A, Emam A, Naguib NN, Nour-Eldin A, Burck I, Stöver T (2015). Pre-, entra- and post-operative imaging of cochlear implants. Rofo..

[17] Rivas A, Marlowe AL, Chinnici JE, Niparko JK, Francis HW (2008). Revision cochlear implantation surgery in adults:indications and results. Otol Neurotol..

[18] Fayad JN, Baino T, Parisier SC (2004). Revision cochlear implant surgery:causes and outcome. Otolaryngol Head Neck Surg.

[19] Zeitler DM, Wang KH, Prasad RS, Wang EY, Roland JT (2011). Flat-panel computed tomography versus multislice computed tomography to evaluate cochlear implant positioning. Cochlear Implants Int.

[20] Bamberg F, Dierks A, Nikolaou K, Reiser MF, Becker CR, Johnson TR (2011). Metal artifact reduction by dual energy computed tomography using monoenergetic extrapolation. Eur Radiol.

[21] Lane JI, Lindell EP, Witte RJ, DeLone DR, Driscoll CL (2006). Middle and inner ear: improved depiction with multiplanar eeconstruction of volumetric CT data. Radiographics..

[22] Reisser C, Schubert O, Forsting M, Sartor K (1996). Anatomy of the temporal bone: detailed three-dimensional display based on image data from high-resolution helical CT: a preliminary report. Am J Otol.

[23] Lane JI, Witte RJ, Driscoll CL, Shallop JK, Beatty CW, Primak AN (2007). Scalar localization of the electrode array after cochlear implantation: clinical experience using 4-slice multidetector computed tomography. Otol Neurotol..

[24] Jäger L, Bonell H, Liebl M, Srivastav S, Arbusow V, Hempel M, Reiser M (2005). CT of the normal temporal bone: comparison of multi- and single-detector row CT. Radiology..

[25] Ishikawa T, Ushiki T, Mizuno K, Togashi T, Watanabe K, Seki K, Ohta H, Yoshida T, Takeda K, Kamimura T (2005). CT-maximum intensity projection is a clinically useful modality for the detection of gastric varices. World J Gastroenterol.

